# Statistical Physics Approach to Liquid Crystals: Dynamics of Mobile Potts Model Leading to Smectic Phase, Phase Transition by Wang–Landau Method

**DOI:** 10.3390/e22111232

**Published:** 2020-10-29

**Authors:** V. Thanh Ngo, Phuong-Thuy Nguyen, Hung T. Diep

**Affiliations:** 1 Center for Informatics and Computing, Vietnam Academy of Science and Technology, 18 Hoang Quoc Viet, Hanoi 10000, Vietnam; nvthanh@cic.vast.vn; 2 Graduate University of Science and Technology, Vietnam Academy of Science and Technology, 18 Hoang Quoc Viet, Hanoi 10000, Vietnam; ntpthuy@iop.vast.ac.vn; 3 Institute of Physics, Vietnam Academy of Science and Technology, 10 Dao Tan, Hanoi 10000, Vietnam; 4 Laboratoire de Physique Théorique et Modélisation, CY Cergy Paris Université (Formerly, University of Cergy-Pontoise), CNRS, UMR 8089, 2 Avenue Adolphe Chauvin, 95302 Cergy-Pontoise, France

**Keywords:** Potts model, Monte Carlo simulation, Wang–Landau method, smectic phase, dynamics, isotropic-smectic phase transition

## Abstract

We study the nature of the smectic–isotropic phase transition using a mobile 6-state Potts model. Each Potts state represents a molecular orientation. We show that with the choice of an appropriate microscopic Hamiltonian describing the interaction between individual molecules modeled by a mobile 6-state Potts spins, we observe the smectic phase dynamically formed when we cool the molecules from the isotropic phase to low temperatures (*T*). In order to elucidate the order of the transition and the low-*T* properties, we use the high-performance Wang–Landau flat energy-histogram technique. We show that the smectic phase goes to the liquid (isotropic) phase by melting/evaporating layer by layer starting from the film surface with increasing *T*. At a higher *T*, the whole remaining layers become orientationally disordered. The melting of each layer is characterized by a peak of the specific heat. Such a succession of partial transitions cannot be seen by the Metropolis algorithm. The successive layer meltings/evaporations at low *T* are found to have a first-order character by examining the energy histogram. These results are in agreement with experiments performed on some smectic liquid crystals.

## 1. Introduction

Since the discovery of liquid crystals (LC) [1,2], many investigations have been carried out to understand their behaviors. This is due to numerous applications such as digital displays that we see in our daily-life objects. Liquid crystals are often made of elongated organic rode-shaped molecules. Due to such strong structural anisotropy molecules and their interaction, these molecules are arranged to form states between liquid and solid with some ordering depending mostly on the temperature but for some mesomorphic phases, the LC ordering can also be a function of the concentration of the molecules in a solvent. The LC phases are often called mesophases, which include four kinds of structure, according to the degrees of symmetry and the physical properties of LC with respect to their molecular arrangement: nematic, smectic, cholesteric and columnar LC.

Among these structures, the smectic phase is almost a crystalline solid for which molecules are ordered in equidistant layers. It shows a long-range positional order and also an orientational order in each layer. We know that the smectic phase, as the other LC phases, has different microscopic origins. Though their orderings belong to the same classification, namely the smectic phase, each smectic LC may have properties different from the others, for example the temperature-dependence of physical quantities and the reaction to an applied electric field. The difference comes from the fact that LC phases are experimentally observed in various materials that have different constituents with different types of interaction. Theoretically, properties of an LC phase are determined from the microscopic model defined by a Hamiltonian. We note the similarity with magnetic systems: for example, ferromagnetic materials have different properties depending on the material, namely the spin model and the interaction type. We know that the transitions for Ising, XY and Heisenberg spin models in ferromagnets belong to different universality classes [3].

We are interested in this paper in the properties of the smectic phase when the temperature varies. In spite of the fact that there has been an enormous number of theoretical and experimental studies over the last 40 years [1], there is only a limited number of reports on the nature of the smectic–isotropic transition. Most of the theoretical studies have used Frank’s free energy [4,5,6], which is a phenomenological macroscopic expression of different mechanisms such as splay constraint, twist and bend distortion, which determine the ordering of a liquid crystal. Numerous models have been developed for modeling LC based on the Frank’s free energy. The Frank’s energy does not, however, describe the dynamics of constituent molecules leading to the formation of an LC. Other approximations using Landau-de Gennes mean-field free energies yield mainly mean-field results.

It is important to note that there has been a large number of theoretical works using the hard-sphere model in which the interaction between molecules is described by a contact potential. These works have been reviewed in Reference [7] where various theories and approximations have been thoroughly discussed. It is noted that these works studied systems at equilibrium using a free energy expanded at most to the second order. The FMT (Fundamental Measure Theories) [8,9] also uses this way of approach with the weighted density approximation. What we said about Landau–de Gennes mean-field theories still applies to this case. Note that the term Density Functional Theory (DFT) used in these works is not the DFT used to indicate first-principle ab-initio calculations using the Kohn–Sham scheme and commercialized packages to treat different potential terms. The DFT used in the above review is to indicate the density functional ρ used in the free energy. The works using hard-body potentials reviewed in [7] gave invaluable insights into different LC structures. As these authors said in their conclusion, though anisotropic hard interactions are enough to explain the ordering in nematic, smectic, columnar LC and many other mesophases, hard-body interactions may not be essentially responsible for the stability of liquid crystalline phases in real materials.

We mention here another family of works on the layer-thinning transitions of freely-suspended smectic films found experimentally and described theoretically. There exists a vast literature on this topic. Let us just mention a few experimental works published in [10,11,12,13] and some theoretical works treating this subject in [14,15,16]. The titles of these references give the information on the studied systems. As seen below, our model belongs to this family of models that studies the melting/evaporation transition of surface layers.

On the computing works, including Monte Carlo simulations and molecular dynamics, there has been a large number of investigations using various models. There have been early numerical simulations on the isotropic-nematic transition [17,18] as well as other works using artificial interactions of molecules-recipient wall [19,20] or approximate free energy [21] for this transition. In a pioneer work, Fabbri and Zanonni [18] considered the Lebwohl–Lasher model [17] of nematogens occupied the sites of a cubic lattice. They interact with each other via a pair potential $U_{i,j}=-\epsilon_{ij}P_{2}\left(cos\beta_{ij}\right)$, where $\epsilon_{ij}$ is a constant for nearest neighbors particles (i,j) and $\beta_{ij}$ is the angle between their molecular axes. This model, which is a Heisenberg spin model localized on a lattice, paved the way for many other simulations in the following 20 years. Let us mention a few important works concerning the nematics. In References [22,23], Monte Carlo simulations have been performed with a generalized attractive-repulsive Gay–Berne interaction, which is derived from the Lennard–Jones potential. In Reference [24], the authors have established by Monte Carlo simulations the phase diagram of a system of molecules interacting with each other via an anisotropic potential. In Reference [25], simulations have been performed on the Lebwohl–Lasher model with the introduction of an amount of spin disorder. We can mention the review by Wilson [26] on the molecular dynamics and the books edited by Pasini et al. [27,28], which reviewed all important computing works. In particular, an excellent review is given in Reference [29] in atomistic simulations and modelings of smectic liquid crystals. We should mention also a numerical work on the nematic transition using Brownian molecular dynamics [30] and a few Monte Carlo works with molecules localized on the lattice sites [31,32].

All the works mentioned above did not take into account the mobility of the molecules in the crystal, therefore they did not show how dynamically the molecules move to form an LC ordering when the system is cooled from the liquid phase. In addition, no simulations have been done to study the isotropic–smectic and isotropic–nematic phase transition taking into account the mobility of the molecules at the transition, in spite of a large number of experimental investigations that will be mentioned below. In addition, no simulations have been done to study the nature of the isotropic–smectic transition. Recently, it has been shown that the smectic and nematic phases can be obtained directly by cooling the system with appropriate Hamiltonians using a mobile Potts model [33]. To our knowledge, this work is the only statistical study of systems of “mobile” interacting molecules, which dynamically leads to the formation of an LC ordering.

This motivates the study presented in this paper. Here, we use the same mobile molecule model as in Reference [33] with appropriate interactions allowing to generate the smectic ordering. Our main objective is to determine the nature of the transitions by using the high-performance of the Wang–Landau flat energy-histogram method [34,35]. The determination of the nature of the phase transition in LC is very difficult, both experimentally and theoretically. Unlike spin lattice models where the phase transitions and critical phenomena have been well studied, molecules in LC have complicated structures to be realistically modeled by a spin language, in addition to the fact that they move in space. A transition in an LC often combines a disordering of molecular orientations and a rearrangement of their positions. There has been, therefore, a small number of such studies in the past. We can mention some important experimental works to show that the phase transition in LC is complex. Z. Dogic has shown the role of the surface freezing in the isotropic–smectic phase transition in a system of colloidal rods [36] Dogic and Fraden [37] have also developed a model colloidal liquid crystals to study the kinetics of the isotropic–smectic transition. They have observed a number of novel metastable structures of unexpected complexity. They have also investigated the smectic phase in a system of semiflexible virus particles [38] and found that a transition to the isotropic phase is of first order. Other complicated experimental observations have been reported [39,40,41,42]. A detailed discussion was given in Reference [43] on the weakly first-order nature of the nematic–isotropic phase transition observed in seven compounds. On the theories, some works mostly based on the Landau theory have been carried out to show that depending on the Hamiltonian, the isotropic–smectic transition is complicated, and can be of first or second order [44,45,46].

In the present paper, we aim at determining the order of the phase transition observed in our LC model described below.

The paper is organized as follows. We will describe the model for the smectic case in Section 2. The six-state Potts model is used to characterize six different spatial molecular orientations. The molecules can move from one site to another on a cubic lattice. Only a percentage of the lattice is occupied by these molecules, the empty sites allow the molecules to move as in a liquid at high *T*. As will be seen, by Monte Carlo (MC) simulations we succeed in obtaining the smectic ordering, by following the motion of molecules with decreasing temperature. This confirms the results obtained in Reference [33]. Our main results are shown in Section 3 where we investigate the nature of the successive layer meltings below the overall smectic–isotropic phase transition by employing the Wang–Landau algorithm. Section 5 is devoted to a conclusion.

## 2. Model

### 2.1. Model

The Hamiltonian used to model the smectic LC is given by

(1) $$H=-\sum_{\langle i,j\rangle }J_{ij}\;\delta_{\sigma_{i}^{},\sigma_{\;j}^{}}$$

where 〈i,j〉 indicates the pair of nearest neighbors (NN). $J_{ij}$ denotes the spin–spin exchange interaction such as

$$J_{ij}=\left\{ \begin{array}{c} J_{//}=J>0,\;\mathrm{in-plane interactions between NN,}\\ J_{⊥}=-aJ<0,\;\mathrm{inter-plane interactions between NN.} \end{array}\right.$$

where a>0. *J* is a constant and will be taken equal to 1, which serves as the energy unit in this paper. The Boltzmann constant is taken as $k_{B}=1$ so that the temperature shown below is in the unit of $J/k_{B}$.

The exchange interactions inside a plane are ferromagnetic and those between neighboring planes are antiferromagnetic. The use of an antiferromagnetic between planes is to avoid a correlation between adjacent planes: The antiferromagnetic interaction favors different spin orientations between NN planes. The Potts spin $\sigma_{i}$ has six values, which represent six spatial molecular orientations. Note that these orientations can be arbitrary with respect to the lattice axes. For example, the six molecular orientations can be 2π×n/6,n=0,…,5 in the xy plane so that $\sigma_{i}$ can take any angle among the six. They can be six orientations in a three-dimensional space where $\sigma_{i}$ is described by two angles $\left(\theta_{n},\phi_{n}\right)$ with n=1,…,6, $\theta_{n}\in \left[0,\pi \right]$ and $\phi_{n}\in \left[0,2\pi \right]$. The Potts model does not need a specification of the value of the molecular orientation: if the NN orientations are similar, the energy is $-J_{ij}$, otherwise the energy is zero.

### 2.2. Formation of the Smectic Phase

The model used in this paper is based on a mobile Potts model with anisotropic interactions given by Equation (1), following Reference [33]. An isotropic mobile Potts model has been used to study the melting of a crystal [47], not in the LC context.

Let us consider a system of $N_{s}$ molecules occupying a simple cubic lattice having $N_{L}$ sites. We consider the situation where $N_{s}\leq N_{L}$. Each site *i* can thus be empty or occupied by a molecule $\sigma_{i}^{}$ having q=6 orientations ($\sigma_{i}^{}=1,2,\ldots ,q$). A molecule can thus go from one site to an empty site by the interaction with neighboring molecules at the temperature *T*. Obviously, the concentration of molecules $c=N_{s}/N_{L}$ must be lower than 1 to permit their motion.

We fix a concentration *c* low enough to allow the motion of molecules inside the recipient. The use of periodic boundary conditions in three directions reduces the size effect. We use several recipient volumes to test the validity of our results and we see that results do not qualitatively change.

To show that the model (1) results in the formation of the smectic ordering, we cool the crystal from high *T*. The simulation is carried out as follows.

To see the dynamics of the molecules upon slow cooling, we use the Metropolis algorithm [48,49]: we generate the positions and the orientations of the molecules randomly in the recipient, we update each molecule’s position and orientation at the same time by comparing the energies of its old and trial new states. The position update is done by moving the molecule to a nearby vacant site with a probability for the simple cubic lattice. The motion of each molecule is therefore driven by just the interaction with its neighbors at a given temperature *T*. We start from a random configuration, namely *from the disordered phase*, and we slowly cool the system with an extremely small interval of *T*.

For the smectic ordering, the natural order parameter is the layer magnetization defined for layer *m* by

(2) $$M_{m}=\frac{1}{q-1}\left[\frac{q}{N_{m}}\;\max_{j\in ⟦1,q⟧}\left(\sum_{i\in \mathrm{layer}\;m}\delta_{j,\sigma_{i}}^{}\right)-1\right]$$

where $N_{m}$ is the number of molecules present on layer *m*. We explain our order parameter defined in Equation (2). This order parameter is defined for each layer separately. We show in the following that $M_{m}$ defined for the layer *m* expresses both the orientational order and the translational order of that layer. We have to examine all lattice layers in order to see the ordering of the positions of the molecules and their orientational ordering. Let us consider the following situations:

1. If a layer *m* is occupied (we count the number of molecules present on each layer) and all $\sigma_{i}$ belonging to the layer *m* have the same orientation, then the sum on delta in Equation (2) should give $N_{m}$, which is the number of molecules present on the layer at the time *t*. So, the quantity in the square bracket is equal to q−1, leading to $M_{m}=1$. This situation corresponds to a layer fully occupied by the molecules of the same orientation. The fact that the lattice of the layer *m* is occupied means that we have the positional order. At the same time we have an orientational order (molecules having the same orientation). This is the case when *T* tends to 0. We have observed a number of layers with such positional ordering and orientational ordering at low *T*. The molecular orientations of these layers are independent of each other. This is the smectic ordering (stop the video shown in Reference [50] at a *T* below 0.3 to see both the positional and orientational orderings).

2. At higher *T*, a number of molecules quit the topmost layer leading to the evaporation of the first layer. With increasing *T*, the second layer will melt, etc. However, the inner layers remain ordered both translationally and orientationally as indicated by our analysis shown in Section 3. The melting of layer by layer into the isotropic state makes the total *M* have a step structure, as seen later in Section 3.

3. If a layer *m* is occupied (we count the number of molecules present on each layer) and $\sigma_{i}$ belonging to layer *m* have all orientations from 1 to *q* (orientation disorder), then the sums inside max(…) give $N_{m}/q$ so that $M_{m}$ is zero as seen by Equation (2). Thus $M_{m}=0$ corresponds to a layer with an orientational disorder. Since the layer is occupied, this case corresponds to the case with translational order but no orientational order. If a layer is empty $M_{m}$ is zero as seen by its definition (since $N_{m}=0$), namely there is neither translational nor orientational order. This is the isotropic phase.

Note that the task of counting the molecules of the same orientation for each layer is done in real time with the simulation.

To summarize, we emphasize that the translational ordering in each of the layers is the first thing to check in the simulation. If a layer is occupied, then we look at the orientational ordering by performing the sum on delta in Equation (2).

We record physical quantities and the motion of molecules as the time evolves. A video showing the dynamics of the formation of the smectic ordering is available at the link given in Reference [50]. Note that the smectic phase at low-temperature consists of planes of different colors, namely different molecular orientations, at equidistance. We observe that the energy curve in the video has several changes of curvature at different temperatures. When the energy changes its curvature, the specific heat goes through a maximum. This may correspond to a phase transition. It is our aim to determine the nature of those maxima and to determine the order of each transition. This is shown in the next section.

## 3. Nature of the Layer Melting/Evaporation

Let us employ in the following the Wang–Landau flat energy histogram method [34,35]. The advantage of this technique is that the density of state (DOS), denoted by g(E), obtained from the simulations, does not depend on the temperature. Using this DOS, one can evaluate the statistical average of a quantity *A* at any temperature *T* given by the formula below

(3) $$\langle A\left(T\right)\rangle =\sum_{E}Ag\left(E\right)e^{-E/k_{B}T}/\sum_{E}g\left(E\right)e^{-E/k_{B}T},$$

where *E* denotes the total energy of the system and $k_{B}$ is the Boltzmann constant. The Wang–Landau technique has been devised for efficiently detecting weak first-order phase transitions.

### 3.1. Implementation of the Wang–Landau Method

The flat energy histogram technique [34,35] relies on an algorithm conceived for classical spin models. This algorithm estimates accurately the density of states g(E). The reader is referred to the original papers [34,35] for details. The efficiency of the Wang–Landau method has been shown in several systems where the nature of the transition has been a controversial subject [51,52,53,54,55,56]. We know that a flat energy histogram H(E) is obtained when the transition probability to the microscopic state of energy *E* is $\propto g{\left(E\right)}^{-1}$. For the details of our implementation the reader is referred to Reference [51]. Let us emphasize that if *E* and $E^{\prime }$ are the initial and final energies in a spin flip, the transition from *E* to $E^{\prime }$ obeys the probability

(4) $$p\left(E\to E^{\prime }\right)=\min\left[g\left(E\right)/g\left(E^{\prime }\right),1\right].$$

The criterion for the energy flatness is

(5) H(E)≥x%.〈H(E)〉

in the considered energy range. 〈H(E)〉 indicates the statistical mean value of H(E). We have fixed x=95% to have good precision.

The total energy *E*, the heat capacity $C_{v}$, the layer magnetization *M* and the susceptibility χ can be evaluated by [34,35,57]

(6) $$\begin{array}{ccc} \langle E^{n}\rangle & = & \frac{1}{Z}\sum_{E}E^{n}g\left(E\right)\exp\left(-E/k_{B}T\right), \end{array}$$

(7) $$\begin{array}{ccc} C_{v} & = & \frac{\langle E^{2}\rangle -{\langle E\rangle }^{2}}{k_{B}T^{2}}, \end{array}$$

(8) $$\begin{array}{ccc} \langle M^{n}\rangle & = & \frac{1}{Z}\sum_{E}M^{n}g\left(E\right)\exp\left(-E/k_{B}T\right), \end{array}$$

(9) $$\begin{array}{ccc} \chi & = & \frac{\langle M^{2}\rangle -{\langle M\rangle }^{2}}{k_{B}T} \end{array}$$

where n=1,2, and the partition function *Z* is given by

(10) $$Z=\sum_{E}g\left(E\right)\exp\left(-E/k_{B}T\right)$$

The canonical probability at any *T* is given by

(11) $$P\left(E,T\right)=\frac{1}{Z}g\left(E\right)\exp\left(-E/k_{B}T\right).$$

Note that in the flat histogram technique we have to choose an energy range $\left(E_{\min},E_{\max}\right)$[58,59]. We divide this energy range to *R* subintervals with overlaps between them to have a smooth matching between two consecutive subintervals. The details of this implementation have been given in Reference [51]. We calculated the relative DOS of each subinterval with the flatness criterion x%=95%. The DOS of the whole energy range is obtained by joining the DOS of each energy subinterval.

### 3.2. Results

We consider an empty simple cubic lattice of $N_{L}$ sites, namely $N_{L}=L_{x}\times L_{y}\times L_{z}$. We fill this empty lattice with $N_{s}$ molecules with $N_{s}\leq N_{L}$. The concentration *c* of molecules in the lattice is defined by $c=N_{s}/N_{L}$.

In our simulations we will take c=30%, 50%, 60%, 80% and 100% so that we can detect the change of the system behavior as a function of *c*. In order to have the same amount of molecules, we keep $N_{s}$ constant ($N_{s}=12^{3}$) in the simulations and take $N_{L}=12\times 12\times L_{z}$ where $L_{z}$ varies from 36 (c=0.3) to 12 (c=1) passing by 24 (c=0.5), 20 (c=0.6) and 15 (c=0.8).

The in-plane and inter-plane interactions between the molecules are taken in this section as $J_{\|}=1.0$ ($J_{\|}$ is taken as the unit of energy) and $J_{⊥}=-0.5$. As said above, the use of an antiferromagnetic between planes is to avoid a correlation between adjacent planes so as to realize the smectic phase. As long as $J_{⊥}<0$, its value is not important.

Using the above-described system, we calculate the DOS by the Wang–Landau method. Once the DOS is obtained, we calculate thermodynamic quantities defined in Equations (6)–(9) and establish the energy histogram with the formulas of canonical distribution [3].

**Figure 1:**
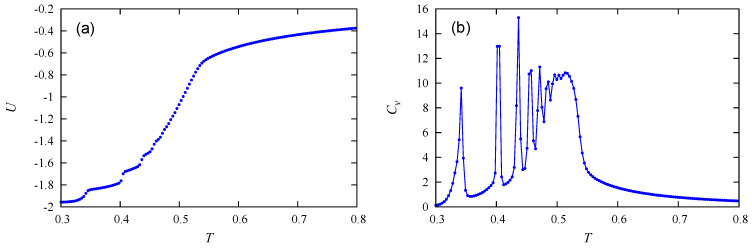
Wang–Landau flat energy histogram results: (**a**) energy per molecule as a function of temperature *T*, (**b**) specific heat vs. *T*. See text for comments.

We display in Figure 1 the energy *U* per molecule and the heat capacity $C_{v}$ obtained by the Wang–Landau method for c=30%. We see nine small changes of curvature of *U* with nine corresponding peaks in $C_{v}$. The last one is the overall phase transition, which occurs at T=0.51099 (in unit of $J_{\|}/k_{B}$). Note that the result obtained by the Metropolis algorithm does not allow us to see the peaks in $C_{v}$ with such precision. As will be shown below, these peaks correspond to the melting of the first, second, third, …ninth layer. The remaining three layers melt at T=0.51099.

**Figure 2:**
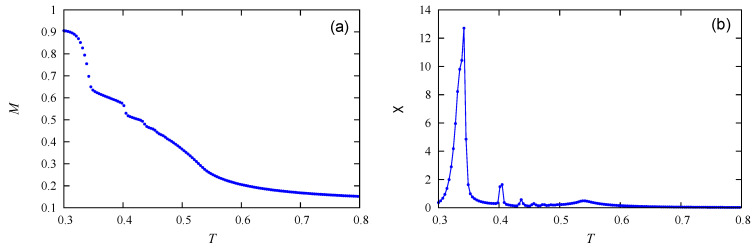
c=30%: (**a**) Total order parameter vs. temperature *T* obtained by Wang–Landau flat energy histogram method, (**b**) susceptibility vs. *T*.

The total order parameter *M* and the susceptibility χ versus *T* are shown in Figure 2. The layer melting mentioned above is seen by the diminution of *M* and peaks of χ.

At this stage, let us describe the scenario of the phase change with varying *T*. We know that melting is the change from solid to liquid phase while evaporation is the change from liquid to gas phase. In our system, at very low *T*, molecules form a layered solid occupying the xy planes due to the in-plane attraction between molecules of the same orientation. At T=0, each of these planes has two neighboring planes containing molecules of different orientation because of $J_{⊥}^{}<0$. Except the case where c=100%, there is an empty space next to the two surfaces of the film. When *T* is increased, it is obvious that molecules on the surfaces move to the empty space, other molecules move on the surfaces to occupy vacant sites. At a constant *T*, the surfaces behave as a liquid with molecules going out or coming back from the free space. We take the case where c=30%. The height of the recipient is $L_{z}=36$. The layered slice at T=0 has 12 planes containing molecules. When we heat the system, two planes on each side of the slice melt/evaporate. This is seen by examining the order parameter at T=0.3418 in Figure 2a where *M* falls to ≃0.65, which corresponds to the disordering of four exterior layers: (12−4)/12≃0.66. We continue to go to T=0.4031: we see that *M* falls to ≃0.5, which corresponds to the melting/evaporating of two more layers, one on each side: we have indeed M=(12−4−2)/12=0.5. It is interesting to note that the empty space contains now molecules in a gaseous state numerous enough to somewhat prevent other molecules of the crystal from evaporating. We see that *M* does not make sharp falls after T=0.4031 although the molecules continue to evaporate, giving rise to the other peaks of $C_{v}$ and χ.

**Figure 3:**
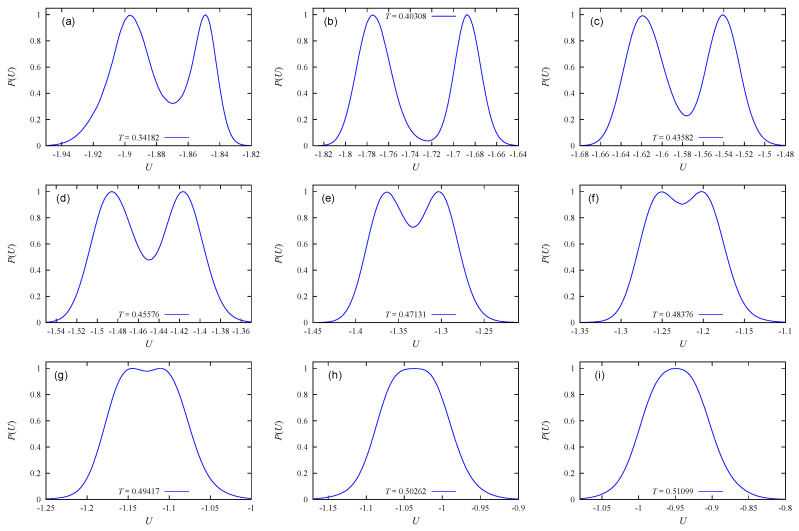
c=30%: Energy histogram at peak temperatures of $C_{v}$ indicated on each histogram. These energy distributions were obtained by the Wang–Landau flat energy histogram method. Single and double peaks indicate second-order and first-order transitions, respectively.

We show now the energy histogram P(U) at the melting of the layers in Figure 3. Though the quantity of molecules is modest, namely 12×12×12, we see the double-peak structures of the energy of eight transitions. The last peak is a Gaussian one. Therefore, the first eight transitions are of first order. The last transition is a second-order transition, as will be explained below.

As mentioned above, the peaks of $C_{v}$ correspond to the successive meltings/evaporations of layers. As we know, melting is always of first order in three dimensions (3D). In our case, the layers melt/evaporate, one after another, also with the first-order character as *T* increases.

Let us take a moment for a discussion. We know that in 2D, atoms can form a crystalline solid at zero temperature. However, when *T* becomes finite, the long-range ordering is destroyed. This can be seen by calculating the displacement of atoms using phonon spectrum at T≠0: it diverges [3,60,61]. The absence of a long-range order is rigorously shown for continuous spin models such as the Heisenberg model with short-range interactions in 2D [62]. In our model, even the system is 3D, the interactions are defined such that in the ground state there is an in-plane ordering but there is no interaction energy in the third direction (that is a characteristic of the smectic phase). When *T* is increased, the system behaves more or less as an assembly of quasi-2D sheets. However, unlike the 2D phonons and 2D continuous XY spins mentioned above, these sheets have a long-range order at T≠0 because of the discrete nature of Potts spins. Note that this long-range ordering is for the case of spins localized on the lattice sites. The 2D sheet of mobile spins does possess a crystalline order at T≠0 but evaporates at a very low *T* compared to the 2D localized spins. We will see below, Equation (12), that the 2D 6-state localized Potts model undergoes a transition at $k_{B}T_{c}=0.8076$ while the 6-state mobile Potts model here melts/evaporates at T≃0.3418 for c=30%. This value should be lower if the empty space is not limited (for higher *c*, namely the empty space is smaller, the melting/evaporating of the first layers takes place at a higher *T*, see below).

At a temperature high enough all remaining layers become disordered altogether. This transition is not a simple melting, but the disordering of still-localized molecules (except molecules at the surfaces). This transition for any *c* is of second-order character. This is not surprising because this transition is the disordering of the localized molecules’ orientations of the remaining core layers, namely the disordering of the Potts model. This transition is not that of the pure 6-state Potts model, but the transition of the effective 4-state Potts model (see the second point of Section 3.3), which is of second order [63,64] (see also discussions on antiferromagnetic Potts models in Reference [65]).

**Figure 4:**
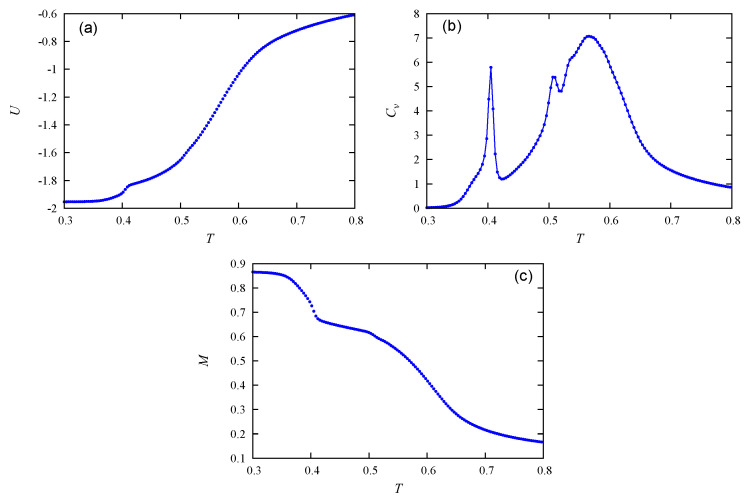
c=50%: Results using the Wang–Landau flat energy histogram technique: (**a**) energy per molecule as a function of temperature *T*, (**b**) specific heat vs. *T*, (**c**) order parameter *M* vs. *T*. See text for comments.

Let us examine now the case of c=50%. Figure 4 displays the energy, the specific heat and the order parameter versus *T*. In this case note that one has less room than the case c=30%. So, one can imagine the evaporation is more difficult. Indeed, one can identify only three peaks of $C_{v}$ and χ. The shoulder before the first peak and before the last peak are not transitions. Again here, the first transition at T=0.4050 corresponds to the melting of four outside layers, two on each side, *M* consequently falls sharply to M=(12−4)/12=0.66 as seen in Figure 4c.

**Figure 5:**
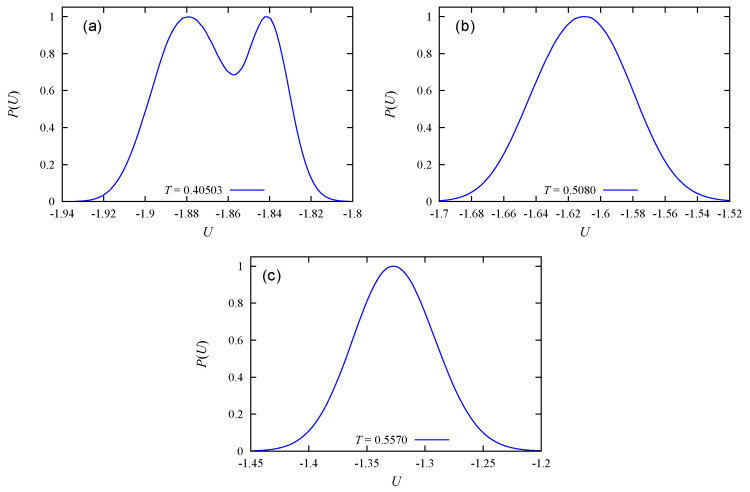
c=50%: Energy histograms by the Wang–Landau flat energy histogram method at the peak temperatures of $C_{v}$. These temperatures are indicated on each sub-figure. Only the first transition at T=0.40503 in figure (**a**) is of first order. Transitions in (**b**) and (**c**) are of second order.

Figure 5 shows the energy histograms at the peak temperatures of $C_{v}$. As seen, only the first transition has the double-peak histogram indicating a first-order transition.

**Figure 6:**
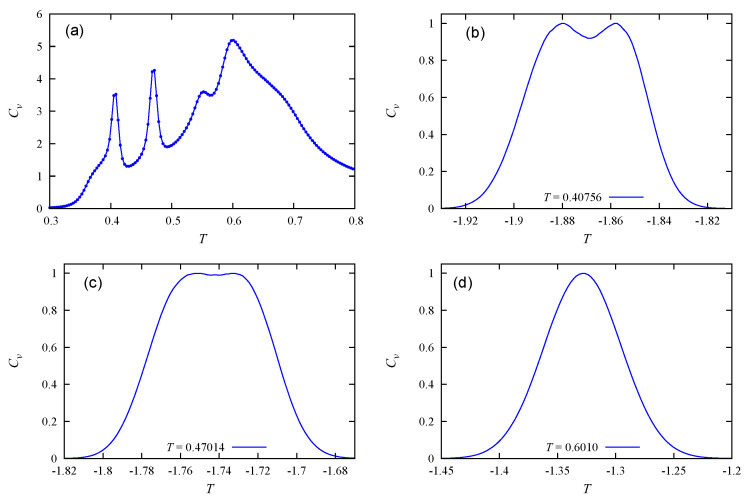
c=60%: (**a**) Specific heat. Energy histogram at (**b**) T=0.40736, (**c**) T=0.47014, (**d**) T=0.6010, by the Wang–Landau method.

Figure 6 shows the energy distributions at the three peaks of $C_{v}$ in the case c=60%. Only the first two peaks correspond to first-order transitions, which occur at T=0.40703 and T=0.47014. Note that the shoulder at T=0.5520 is not a transition.

**Figure 7:**
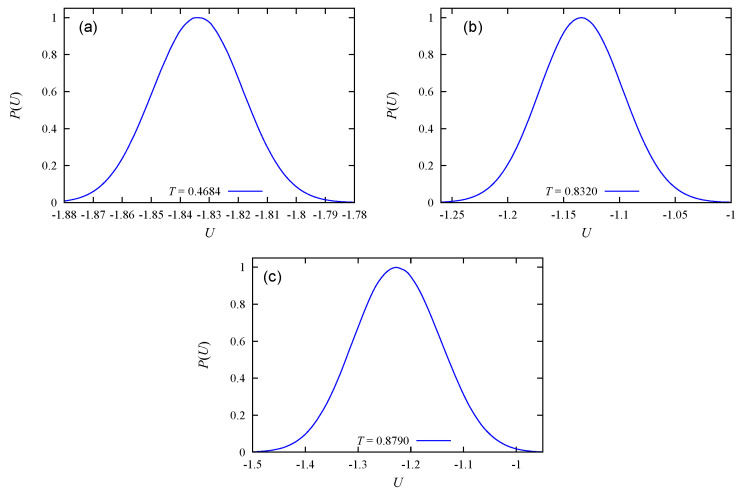
Energy histogram at the peak temperatures indicated on each sub-figure, obtained by the Wang–Landau flat energy histogram method: (**a**) and (**b**) c=80%, (**c**) c=100%: See text for comments.

As noticed earlier, when there is less empty space for evaporation, the transition becomes of second order. We observe this in Figure 7 where the energy distributions indicate second-order transitions for c=80% and 100%.

### 3.3. Discussion

**Figure 8:**
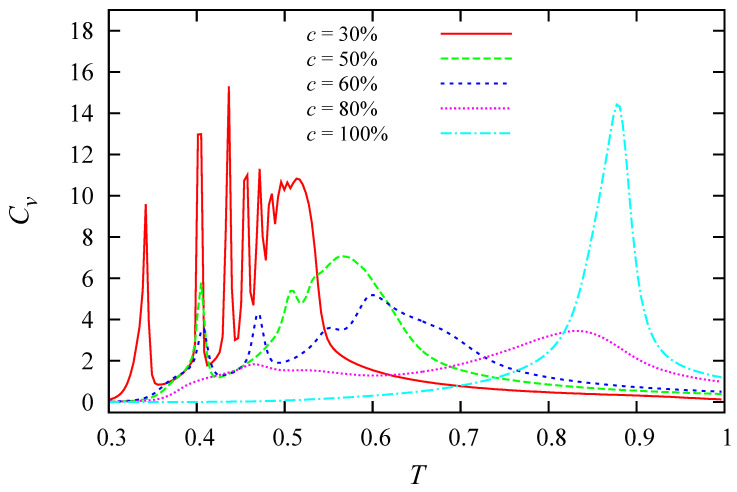
The specific heat vs. temperature by the Wang–Landau method for several concentrations: 30% (red), 50% (green), 60% (blue), 80% (magenta) and 100% (azure).

To end this section let us show the effect of the concentration in Figure 8. We emphasize the following points:

(i) The number of layers that melt at low *T* increases with decreasing concentration (see the curve for 30% for example). This is due to the fact that there is more empty space for a molecule to move at a lower concentration, leading to melting of more layers.

(ii) At 100%, due to the antiferromagnetic interaction in the *z* direction, the molecular orientations of the neighboring planes are different from each other in the ground state. At finite *T*, due to thermal excitations, molecules between adjacent planes are coupled: when their orientations are similar, the energy is higher because $J_{⊥}<0$, see Equation (1). We understand thus that a molecule cannot be excited to the states of its neighbors in the adjacent planes due to the penalty of an energy increase. This mechanism retards the phase transition. A molecule in a plane has two NN molecules in the neighboring planes. Due to the inter-plane antiferromagnetic coupling, these neighboring planes have two different molecular orientations different from the plane under consideration. Thus, the molecule under consideration has to choose one among the four remaining orientations. This case corresponds to the 2D 4-state Potts model. So, we conjecture that the model of this paper is an effective 4-state model (if the NN planes have the same orientation, then the molecule under consideration can take one of the five remaining states, but the probability for this case is 1/36, very small with respect to the case where two planes have different orientation). In other words, our 3D model approximately behaves as a 2D 4-state Potts model. To check this conjecture, let us consider the *q*-state Potts model in 2D. The exact transition temperature is given by [63]

(12) $$k_{B}T_{c}={\left[\ln(1+\sqrt{q})\right]}^{-1}$$

For q=6, one has $k_{B}T_{c}=0.8076$, for q=5 one has $k_{B}T_{c}=0.8515$, and for q=4, one has $k_{B}T_{c}=0.91024$. The peak temperature $T_{c}=0.8750$ found in Figure 8 for the concentration of 100% is between the 4-state model and the 5-state model. It is closer to the 4-state model if we increase the lattice size ($T_{c}$ is increasing with increasing size, see Reference [47]). The qualitative argument we give above explains well the MC result.

To summarize, we show in Figure 8 the specific heat for all concentrations that have been studied. Note that some shoulders are not transition peaks as we have discussed above.

Let us give in the following a discussion closer to experiments. As said in the Introduction, experimental systems are very different ranging from biological ones such as semi-flexible virus particles [38], to colloidal rods [36,37], passing by liquid-crystalline polyethers [42] and chemical macro-molecules “Dodecylcyanobiphenyl (12CB)" [39,40,41]. We mention also the analysis on seven chemical compounds MBBA, 5CB, 8CB, 5NCS, 5CN, 8CHBT, and D7AB, with focus on the nature of the observed phase transitions (see Reference [43]). From the theory of phase transitions [3], we know that the order of the transition is governed by the interaction, the symmetry of the order parameter and the space dimension. Experimental works as the ones mentioned above have been performed on different kinds of LC, making it impossible to have a universal model that verifies all observations. The theoretical situation in the literature is very disperse as we mentioned in the References [17,19,20,21,30,31,32,33,44,45,46]. The domain of phase transitions in liquid crystals has not been very theoretically developed as seen in the works mentioned in these references. In particular, the absence of microscopic models with mobile molecules renders difficult the comparison with experiments. Our present work is so far the only statistical study of a system of “mobile" molecules, which dynamically leads to the formation of a LC ordering in successive steps. As shown below, our results are in agreement with a number of experimental observations on the nature of the smectic-isotropic phase transition.

Let us compare our results to experiments. First, in the LC of semi-flexible virus particles, it was found that [38] “flexibility drives the transition first order”. Our model of rigid Potts spin orientations gives rise precisely to a first-order transition in agreement with this experimental case. Second, in another experiment performed on dodecylcyanobiphenyl (12CB) [39], the authors have shown that a strong first-order transition from the smectic A phase to the isotropic one, while the nematic–isotropic transition is only very weakly first order [43]. This indicates that the orientational order in the smectic-A phase is much higher than that in the nematic phase. The authors also stated that in a range of temperature their “result shows clearly the influence of the smectic-A phase on the pretransitional behaviour and together with DSC (differential scanning calorimetry) and low angle X-ray measurements suggests the existence of smectic A type cybotactic groups in the isotropic phase”. Other experiments revealed almost the same situation: [40]. These experimental results correspond to what we found: first-order phase transition and the coexistence of the isotropic phase and the smectic phase (see comments on Figure 1 and Figure 2).

Note that a simple theory based on the Landau free energy has been carried by Mukherjee et al. [44]. They found a first-order transition as we find here but without the dynamics leading to the smectic ordering and without the coexistence of the smectic and isotropic phases. Our results described in this paper, therefore, give all the details of how the partial transition (melting layer by layer) occurs before the overall transition.

Finally, note that the present model is defined on a lattice (grid). In spite of the fixed grid, our molecules are mobile, they are free to move every where in space. We believe that the general aspect of the results presented above remains in the continuous space, as we have seen in lattice models for fluid flows. This is certainly an effect to be checked in the future.

Since there are many kinds of symmetry of LC, the high-performance method used in the present work will be extended to the cases of 3, 4, 5, … molecular orientations, as well as to continuous XY and Heisenberg spin models to treat the case of continuous molecular orientations in smectics.

## 4. Size Effects—The Case of Ferromagnetic Inter-Layer Interaction

**Figure 9:**
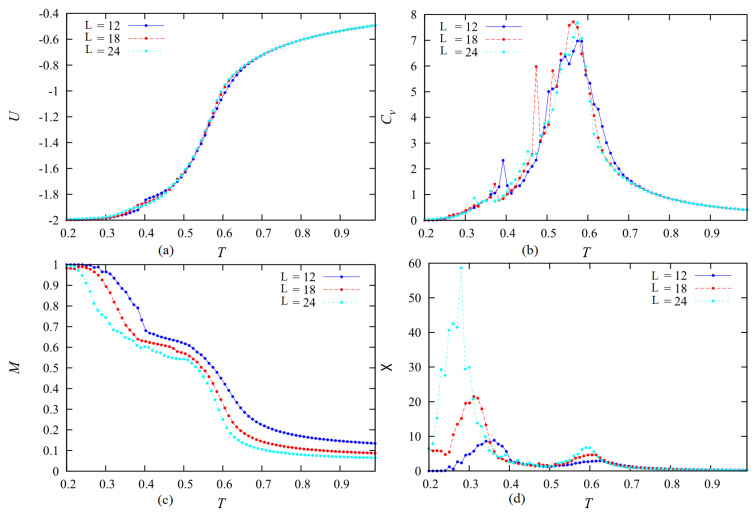
(**a**) The energy, (**b**) specific heat, (**c**) layer magnetization and (**d**) susceptibility vs. temperature at c= 50% with L=12,18,24 (blue, red, green) where $L=L_{x}=L_{y}$. See comments in the text.

Let us touch upon the question of finite-size effects on our results. It is known that the characteristics of a phase transition in systems of particles localized on lattice sites with periodic boundary conditions obey the finite-size scaling laws (see [3] and references therein). However, little is known for spatial translation-broken systems as in the present model. We performed MC simulations for various lateral sizes $\left(L_{x},L_{y}\right)$ at each concentration to examine the size effect. As said in Section 2.2, we used several recipient volumes to test the validity of our results and we have seen that our results do not qualitatively change. Let us show an example for c=0.5 in Figure 9. We observe that the layer melting occurs for three sizes (see the total order parameter in Figure 9c) but the system sizes change the layer-melting transition temperature at low *T* (Figure 9c,d). The internal energy and the specific heat, on the other hand, do not significantly change (Figure 9a,b).

**Figure 10:**
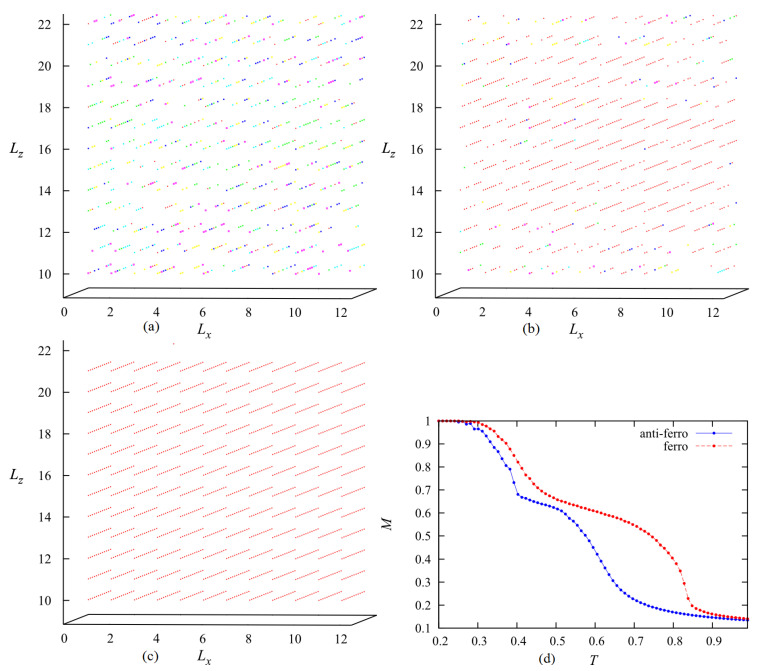
(**a**) Snapshots in the case of ferromagnetic inter-layer coupling $J_{⊥}=+0.5$, (**a**) at high *T* ($T=0.949>T_{c}$), (**b**) at intermediate *T* after a partial surface melting T=0.747, (**c**) at very low *T* (T=0.0251) near the ground state, (**d**) total layer magnetization is shown in the two cases: ferromagnetic (red) and antiferromagnetic (blue) inter-layer couplings, for comparison. Each color represents a molecular orientation. See text for comments. c=50% with $L_{x}=L_{y}=12$.

We discuss now on the case when the inter-layer interaction $J_{⊥}$ is ferromagnetic ($J_{⊥}>0)$, namely when there is an attraction of molecules of the same orientation. Simulations have been performed for $J_{⊥}=+0.5$. In spite of the fact that each molecule has six orientations, the system when cooled down from the isotropic phase reaches a state in which all molecules have the same orientation. Figure 10a–c show the snapshots taken at a high *T*, at an intermediate *T* and at a low *T*. The last figure at low *T* corresponds to a smectic ordering where there is a global orientation. Figure 10b shows the disordering of some outer layers. Figure 10d shows the layer order parameter for both the ferromagnetic and antiferromagnetic cases for comparison. The former case gives a higher transition temperature, but both of them show a plateau at the intermediate temperature region indicating the melting of some outer layers.

## 5. Conclusions

We have considered molecules moving in three dimensional *recipients* with periodic boundary conditions in all directions. In this paper, we have shown that by choosing appropriate interactions for the Hamiltonian of interacting six-orientation molecules, we can get the smectic ordering at low *T*: it suffices to take a strong in-plane ferromagnetic interaction and an antiferromagnetic interaction in the *z* direction and we will get the smectic phase when the system is cooled from the isotropic phase. We have seen at low *T* the molecules gather in independent planes constituting a smectic structure.

So far, there have not been theoretical investigations using a microscopic Hamiltonian such as that given by Equation (1). Most of the theoretical calculations have been based on phenomenological models derived from the Frank’s free energy, which did not allow us to follow dynamically the formation of the LC phase while decreasing the temperature as what we have shown in this paper.

Now if the temperature is increased from T=0, the outside layers melt/evaporate one after another with increasing *T*. By using the high-performance Wang–Landau flat energy-histogram method, we have studied the nature of these layer meltings/evaporations at low *T* and found that they are of first order in agreement with experiments. At high concentrations (80–100%), the smectic-to-isotropic transition is shown to be of second order. This transition is mostly due to the disordering of molecular orientations of the core layers because there is not enough empty space for the molecule mobility. This is similar to what has been observed in the case of the solid–liquid transition in a 3D mobile Potts model (see page 042160-6 of Reference [47]).

As said above, the nature of the transition and the thermodynamic properties of a liquid crystal depends on the interaction between molecules and the spin model for molecules. We have chosen in this work the mobile six-state Potts model for six spatial orientations of molecules and we have used an appropriate Hamiltonian to describe the formation of the smectic phase. However, our method can be extended to other spin models such as the Heisenberg and XY spins and other Hamiltonians to explore various kinds of orderings in liquid crystals.
